# Targeting mitochondrial dysfunction in lung diseases: emphasis on mitophagy

**DOI:** 10.3389/fphys.2013.00384

**Published:** 2013-12-26

**Authors:** Angara Sureshbabu, Vineet Bhandari

**Affiliations:** Division of Perinatal Medicine, Department of Pediatrics, Yale University School of MedicineNew Haven, CT, USA

**Keywords:** mitochondria, reactive oxygen species, mitophagy, apoptosis, pulmonary disease

## Abstract

During mild stressful conditions, cells activate a multitude of mechanisms in an attempt to repair or re-establish homeostasis. One such mechanism is autophagic degradation of mitochondria or mitophagy to dispose damaged mitochondria. However, if stress persists beyond recovery then dysfunctional mitochondria can ignite cell death. This review article summarizes recent studies highlighting the molecular pathways that facilitate mitochondria to alter its morphological dynamics, coordinate stress responses, initiate mitophagy and activate cell death in relevance to pulmonary pathologies. Thorough understanding of how these signaling mechanisms get disrupted may aid in designing new mitochondria-based therapies to combat lung diseases.

## Introduction

Mitochondria are commonly referred to as cellular power centers and perform many biochemical functions ranging from energy production to programmed cell death (Nunnari and Suomalainen, 2012). Mitochondria are large biosynthetic machines responsible for a variety of metabolic (both catabolic and anabolic) reactions including amino acids, lipids and ketone bodies. There are four mitochondrial compartments: the outer mitochondrial membrane (OMM), intermembrane space (IMS), inner mitochondrial membrane (IMM) and the matrix with inner membrane folds called cristae. The most prominent role for mitochondria is to generate NADH and ATP through the tricarboxylic acid cycle (TCA) and oxidative phosphorylation (OXPHOS) (Dromparis and Michelakis, 2013). Mitochondria regulate cellular homeostasis through four factors (1) Membrane potential (2) Mitophagy (3) making Acetyl CoA and (4) Reactive oxygen species (ROS) generation.

Mitochondrial electron transport chain (ETC) comprises five large protein complexes embedded across the IMM responsible for OXPHOS system. These ETC complexes are termed the Complex I (NADH-Coenzyme Q oxidoreductase); Complex II (succinate-Coenzyme Q oxidoreductase); Complex III (Q-cytochrome c oxidoreductase); Complex IV (Cytochrome c oxidase); Complex V (ATP synthase) respectively (Smeitink et al., 2001). The oxidation of nutrients (simple sugars) occurs in a concerted series of subsequent reactions, so that the ATP generated, resulting by the reduction in the electron potential energy, is gradually released. During this process, protons are pumped from the matrix into the IMS leading to the elevated negative charges in the matrix. This is referred to as mitochondrial membrane potential (Δψm). The healthy pool of mitochondria is characterized by high/intact Δψm. Conversely, an unhealthy pool of mitochondria is characterized by low/lost Δψm (Nunnari and Suomalainen, 2012).

## Mitochondria and redox regulation

Jensen first observed the formation of hydrogen peroxide (H_2_O_2_; ROS) in antimycin-insensitive NADH and succinate oxidation reactions (Jensen, 1966). During OXPHOS, superoxide anion radicals (O^−^_2_) are produced predominantly at complex I and complex III as by-products. These superoxide anions are converted into H_2_O_2_ in the matrix with the action of Manganese superoxide dismutase (MnSOD). Superoxide anions, H_2_O_2_ and hydroxyl radicals together constitute ROS (Glasauer and Chandel, 2013). Mitochondria are the major sources and targets of ROS generation, the other being the NADPH oxidases. Abnormal levels of ROS have been implicated in several diseases including but not limited to pulmonary diseases, cardiovascular diseases and gastro-intestinal diseases. However, low concentrations of ROS are important determinants required for physiological signaling of various developmental pathways (Rehman, 2010; Sena and Chandel, 2012).

Mitochondria are typically viewed as oxygen sensors as they respond to low and high concentrations of oxygen. In relevance to this, ROS production determined by mitochondria can be influenced by various oxygen concentrations (Sarsour et al., 2009). ROS can cause cellular damage by oxidizing nearly all the biomolecules including proteins, DNA (both nuclear and mitochondria), lipids and carbohydrates (Handy and Loscalzo, 2012). Oxidative damage to mitochondrial DNA by ROS results in the synthesis of defective ETC subunits, further resulting in the abnormal emission of ROS (Harman, 1972; Miquel et al., 1980). This reflects disturbances in the normal redox balance and manifests the disparity between the prooxidants and antioxidants inside the cell termed as oxidative stress. The overall redox status of the cell is balanced by antioxidant enzymes including glutathione peroxidase, MnSOD, catalase, peroxiredoxin and thioredoxin systems (Li et al., 2013). In addition to these antioxidant enzyme systems, redox status of the cell is further regulated by Δψm and mitochondrial dynamics.

## Mitochondrial dynamics

Mitochondria are dynamic intracellular organelles that constantly change in shape, size, number and distribution through constitutive cycles of fusion and fission (Westermann, 2010). Mitochondrial dynamics are influenced through signaling between the OMM and mitochondria-associated membranes (Nunnari and Suomalainen, 2012). Understanding mitochondrial dynamics is essential to knowing underlying biology as they regulate mitochondrial morphology, mitophagy, apoptosis and other biological functions of mitochondria. Mitochondrial fusion events are known to play a role in maintaining intact mitochondrial DNA copies, mitochondrial membrane components and matrix metabolites (Berman et al., 2008; Twig and Shirihai, 2011). Coordination of double membrane mitochondrial fusion mechanisms has not been fully understood. Multiple lines of evidence suggest that mitochondrial fusion involves mitofusins MFN1 and MFN 2 (OMM proteins) together with optic atrophy protein 1 (OPA1; an IMM protein) (Hoppins et al., 2007). GTPase activity is essential for these three proteins to mediate mitochondrial fusion events (Liesa et al., 2009). Opa1 protein, both short and long isoforms were reported to participate in mitochondrial fusion and protection from apoptosis (Arnoult et al., 2005; Song et al., 2007).

Mitochondrial fission events are known to play role in segregation of dysfunctional mitochondria from the pool of mitochondria (Twig and Shirihai, 2011). The most studied proteins that regulate mitochondrial fission are dynamin-related protein 1 (DRP1) and fission protein 1 homolog (FIS1) (Liesa et al., 2009). Mitochondrial-anchored protein ligase (MAPL) conjugates small ubiquitin-like modifier (SUMO) to DRP1 to activate mitochondrial fission (Braschi et al., 2009). Mitochondrial fission is regulated by DRP1 which translocates to mitochondria by an adaptor protein mitochondrial fission factor (MFF) (Smirnova et al., 1998).

In mammalian cells, preventing mitochondrial fusion or fission contributes to OXPHOS deficiencies, mitochondrial DNA loss and significant generation of excessive ROS (Chen et al., 2003; Chen and Chan, 2004; Meissner, 2007). When the Δψm is low, mitochondrial fission prevails and daughter mitochondria are more prone to mitophagy. Similarly, mitochondrial fission is prevalent in diseased cells with subsequent elimination of damaged mitochondria via mitophagy (Kubli and Gustafsson, 2012). Interestingly, mitochondrial fission occurs early both in the mitophagic and apoptotic pathways. However, mitochondrial fission is not completely required to trigger the downstream pathways. Conversely, it is well established that mitochondrial fusion inhibits apoptotic cell death (Westermann, 2010). Therefore, mitochondrial dynamics are intrinsically linked with the quality control mechanisms, mitophagy and cell death to maintain mitochondrial homeostasis.

## Mitophagy

Mitochondrial autophagy (also referred as mitophagy) is an evolutionarily conserved homeostatic process by which the cell selectively degrades only damaged mitochondria within autolysososmes (Youle and Narendra, 2011). It is well documented that mitophagy is generally inititated when Δψm is low (Kubli and Gustafsson, 2012). Mitochondrial clearance is a highly regulated process and intimately linked with mitochondrial fission and fusion proteins. However, to what extent mitochondrial dynamics influence mitophagy is a subject of intensive research.

**Figure 1:**
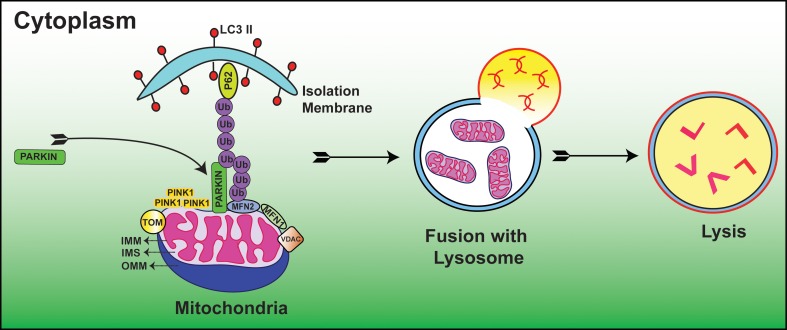
**Diagrammatic representation showing the molecular events of Parkin/PINK1 mediated mitophagy process.** Low Δψm suppresses the activity of TOM complex and leads to the accumulation of PINK1. This is followed by translocation of Parkin from cytoplasm to mitochondria where it is activated by PINK1. Subsequently, Parkin ubiquinates OMM proteins including but not limited to MFN1, MFN2, VDAC, and MIRO-1. These ubquitinated proteins are then recognized by autophagy proteins P62 and LC3 II where subsequent degradation of damaged mitochondria occurs within the mitochondrial membrane; IMS, Inter mitochondrial space; OMM, Outer mitochondrial membrane. (For additional details and abbreviations, please see text).

Autophagic vacuole formation with sequestrated mitochondria was first examined in glucagon stimulated hepatocytes using electron microscopy (Deter and De Duve, 1967). In a seminal study, depolarized mitochondria were found to co-localize with lysosomes in cultured rat hepatocytes treated with glucagon in the absence of serum (Rodriguez-Enriquez et al., 2009). Since then, considerable progress has been made to delineate the molecular mechanisms of mitophagy. Recent studies suggested that E3 ubiquitin ligase, Parkin/PARK2 and phosphatase and tensin homolog (PTEN)-induced putative protein kinase 1 (PINK1) act as master regulators in the elimination of abnormal mitochondria (Dagda et al., 2009; Chu, 2010; Gottlieb and Gustafsson, 2011). In this process, Parkin ubiquinates OMM proteins including MFN1, MFN2 (Chen et al., 2003; Gegg et al., 2010), voltage dependent anion channel (VDAC) (Geisler et al., 2010) and mitochondrial rho GTPase 1 (MIRO-1) (Wang et al., 2011) resulting in autophagic degradation and subsequent alteration of mitochondrial dynamics. When the Δψm is high i.e., under normal steady state conditions, PINK1 is cleaved and imported by the mitochondrial preprotein translocases of the outer membrane (TOM) complex and hence PINK1 is usually found at low levels on mitochondria. Whereas, when the Δψm is low i.e., under stressful conditions, PINK1 accumulates on the OMM and TOM complex loses the ability to recruit Parkin (Lazarou et al., 2012). Furthermore, numerous lines of evidence have demonstrated that accumulation of PINK1 is essential for the translocation and activation of Parkin from cytosol to mitochondria (Kubli and Gustafsson, 2012) (Figure 1). An open question in this field is what regulators guide the recruitment and activation of Parkin. A recent paper reported that PINK1 phosphorylates MIRO-1 and activates proteosomal degradation of MIRO-1 in a Parkin dependent manner (Wang et al., 2011). In agreement with this, PINK1/Parkin pathway sequesters the damaged mitochondria from the healthy ones and thus maintains cellular homeostasis. A recent study clearly demonstrated that Ras homolog enriched in brain protein (Rheb), localizes to the OMM and contributes to mitochondrial degradation (Melser et al., 2013). The authors also showed that Rheb regulates OXPHOS dependent mitophagy. In a recent study, cardiolipin found in the IMM had LC3 binding sites, and when these sites mutated, suppressed its contribution during mitophagy. The authors also showed that cardiolipin is externalized in response to mitochondrial injury and serves as a molecular pattern recognition signal where the autophagy machinery recognizes it and undergoes mitophagy (Chu et al., 2013). Taken together, these studies suggest that cells have evolved different mechanisms to eliminate malfunctioning mitochondria. Thus, mitophagy prevents healthy cellular networks from mitochondrial dysfunction by sequestering the damaged mitochondria and when this fails, mitophagy acts as a prelude to cell death. However, there are still considerable gaps in understanding the molecular signaling of mitophagy in both developmental and diseased conditions.

## Mitophagy regulates ROS levels

Apart from maintaining cellular homeostasis, mitophagy also plays an important role in response to cellular stress (Kroemer et al., 2010; Levine et al., 2011). Depending upon the requirement of levels of cellular ATP, mitophagy regulates mitochondrial quantity. Furthermore, mitophagy regulates excessive mitochondrial ROS by separating toxic or damaged mitochondria from the intracellular networks. Thereby, mitophagy balances the normal redox state of the cell.

A recent paper described that moderate levels of ROS induces mitophagy in a mitochondrial fission dependent manner (Frank et al., 2012). The authors of this study also showed that dominant-negative variant of the fission factor DRP1 inhibited mitophagy. Therefore, it is conceivable that mitochondrial ROS and mitophagy form a feedback loop mechanism whereby mitochondrial ROS and mitophagy regulate each other (Sena and Chandel, 2012).

## Mitochondria and cell death

Failure in the balance of previously described processes triggers mitochondrial dysfunction, and when not repaired, may lead to cell death. Therefore, collapse of Δψm is considered as a cellular death precursor. Numerous lines of evidence have demonstrated that mitochondria regulate three forms of cell death. (1) Extrinsic apoptosis (2) Intrinsic apoptosis (3) Necrosis.

Extrinsic apoptosis is initiated through ligation of death receptors such as tumor necrosis factor receptor 1 (TNFR1) or Fas resulting in the recruitment of a multiprotein complex with caspase 8, receptor-interacting serine/threonine-protein kinase 1 (RIPKI) and Fas-associated death domain (FADD). This leads to the dimerization and activation of caspase 8 inducing the downstream apoptotic pathway. This extrinsic pathway is also referred to as death receptor-induced apoptosis initiated by Fas ligand (FasL), tumor necrosis factor (TNF) or TNF-related apoptosis-inducing ligand (TRAIL) (Tait and Green, 2010).

Loss of integrity in the OMM is considered as a consequence of DNA damage or endoplasmic reticulum stress. Intrinsic apoptosis is triggered by mitochondrial outer membrane permeabilization. This is followed by the subsequent release of several pro-apoptotic factors through mitochondrial permeability transition pores (mPTP). During this process, toxic proteins including but not limited to cytochrome c, apoptosis inducing factor (AIF), second mitochondria-derived activator of caspase/direct inhibitor of apoptosis-binding protein with low pI (SMAC/DIABLO) activate caspase proteases in the cytoplasm leading to cell death (Galluzzi et al., 2012).

In the absence of caspase activity, cell can still undergo death via necrosis or necroptosis. Also ignited by TNFR1, necroptosis occurs through IMM. During this process, RIPK1 and RIPK3 interact with mixed lineage kinase domain-like (MLKL) to form a necrososome. This necrosome is a multiprotein complex that inhibits mitochondrial protein adenine nucleotide translocase (ANT), by reducing glutaminolysis and thereby promoting mitochondrial fragmentation (Tait and Green, 2010). All the three forms of cell death are tightly regulated by mitochondria highlighting a potential therapeutic target. The next section describes mitochondrial dysfunction in various lung diseases.

## Mitochondrial dysfunction in neonatal lung diseases

**Figure 2:**
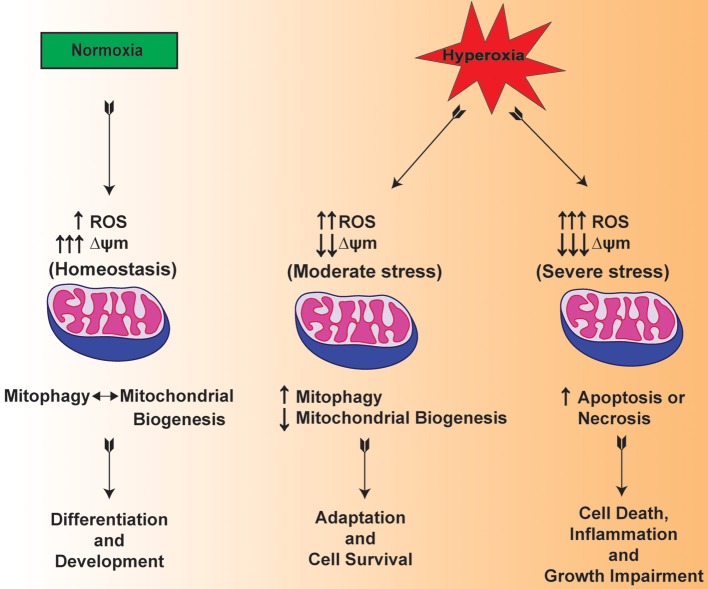
**Hypothetical mechanism illustrating the mitochondrial function in normoxia and mitochondrial dysfunction in hyperoxia exposed lungs of premature infants**.

Mitochondria are one of the main organelles that are significantly impacted by the clinical procedures for the resuscitation of premature infants (Berkelhamer et al., 2013). In this regard, there are recent studies suggesting that mitochondrial dysfunction is considered among the pathogenic factors in the scenario of both mechanical ventilation and hyperoxia exposure (Morton et al., 1998; Ratner et al., 2009, 2013). Multiple lines of evidence suggest that hyperoxia increases the levels of ROS within the mitochondria of lung both *in vitro* and *in vivo* (Freeman and Crapo, 1981; Farrow et al., 2010, 2012) (Figure 2). Of note, both hyperoxia and mechanical ventilation are considered as major risk factors that are associated with developmental alveolar and vascular impairment—the hallmarks of BPD in premature infants (Bhandari and Bhandari, 2007). In a BPD model, mitochondrial aconitase activity was significantly decreased after exposure to 100% oxygen for 6–10 days in the lungs of premature baboons (Morton et al., 1998). BPD is also mediated by the exaggerated increase in the pro-inflammatory cytokines such as interleukin-1β and TNF-α in the bronchoalveolar lavage fluid (Ambalavanan et al., 2009; Sun et al., 2013). In response to hyperoxic exposure, mitochondria showed significantly lowered enzymatic activity of Complex I indicating impaired OXPHOS in newborn mice lungs (Ratner et al., 2009). The data presented in these studies indicate that there is a cross talk between mitochondrial dysfunction and inflammation.

In a recent study, mitochondrial DNA defects caused by hyperoxia dictated the impaired branching morphogenesis and reduced surfactant C expression in fetal rat lung explants (Gebb et al., 2013). Another study reported that reduced levels of MnSOD expression and activity led to endothelial dysfunction caused by oxidative stress in persistent pulmonary hypertension of the newborn in the lamb model (Afolayan et al., 2012). These data indicate that the impaired scavenging of ROS by low activity of antioxidants results in impaired vasodilation in the pathogenesis of pulmonary hypertension. In an elegant study, the ratio of mitochondrial matrix oxidative stress to antioxidant enzymes was noted to be more in neonatal lung slices as compared with adult lung slices with hyperoxia exposure. Further to this, they demonstrated that nicotinamide adenine dinucleotide phosphate dependent oxidase 1 (NOX1) expression was increased in the postnatal day 7 lungs compared with adult lungs during acute hyperoxia (Berkelhamer et al., 2013). These studies reinforce the notion that malfunctioning mitochondria in conjunction with excessive ROS may play a key role in neonatal lung diseases.

## Mitochondrial dysfunction in chronic obstructive pulmonary disease (COPD)

COPD is linked with increased mitochondrial ROS production, decreased anti-oxidant capacity, impaired OXPHOS and reduced mitochondrial number (Kirkham and Barnes, 2013; Meyer et al., 2013). Since the first evidence demonstrating mitochondrial dysfunction in diaphragmatic muscles of COPD patients, multiple lines of evidence suggested that significant skeletal muscle mitochondrial dysfunction occurred among COPD patients (Lloreta et al., 1996; Meyer et al., 2013). Thus, abnormal mitochondria emerged as key players in limb and skeletal muscle impairment in COPD patients.

It is generally acknowledged that cigarette smoke is the major risk factor for COPD. Cigarette smoke extract increases oxidative stress due to impaired mitochondria structure and function in both proximal and distal parts of lung in COPD patients. (van der Toorn et al., 2007, 2009). Expression of higher oxidative stress proteins in concert with decreased expression of antioxidant proteins may thus reflect a state of redox imbalance in COPD. This is caused by the presence of a large number of oxidants (approximately 4700 chemicals) in cigarette smoke. In line with this, multiple lines of evidence showed increased superoxide anion production contributing to skeletal muscle loss and dysfunction in COPD patients, as compared to healthy subjects (Marin-Corral et al., 2009; Barreiro et al., 2010). Another noteworthy observation is that significantly decreased expression of prohibitins namely PHB1, but not PHB2, in COPD and non-COPD smokers as compared to non-smokers correlates with mitochondria dysfunction. This indicated that PHB1 is a fundamentally important membrane protein for maintaining mitochondrial protein stability and function (Soulitzis et al., 2012). A recent study demonstrated mitochondrial fragmentation in cigarette smoke induced COPD model systems *in vivo* (Hara et al., 2013). Taken together, these studies suggest that mitochondrial dysfunction is a key contributor to the pathophysiology of COPD.

## Mitochondrial dysfunction in lung cancer

Mitochondrial DNA (mtDNA) instability has been reported in various human cancers including lung cancer (Chatterjee et al., 2006; Yang Ai et al., 2013). In a recent study, increased mtDNA copy number suggested an elevated risk of lung carcinogenesis. In another study, presence of significant high copy number of mtDNA mutations in respiratory complex-I of OXPHOS system in “never smokers,” as compared to current smokers, lung cancer patients was reported. Furthermore, mtDNA mutation content was significantly associated with epidermal growth factor receptor (EGFR) gene mutation (exons 19 and 21) in “never smokers” lung cancer patients (Dasgupta et al., 2012). In parallel, tumor suppressor genes such as p53, Ras, and Myc are known to be highly sensitive to mitochondrial ROS resulting in their activation and ultimately guide the progression of inflammation associated cancer. These studies suggest that both mtDNA mutations and nuclear DNA mutations signal through mitochondrial dysfunction and contribute to the progression of lung cancer (Kamp et al., 2011).

## Mitochondrial dysfunction in asthma

Asthma is a heterogeneous chronic inflammatory disease characterized by variable airway obstruction, airway remodeling and bronchial hyper-responsiveness (Reddy, 2011; Lambrecht and Hammad, 2012). Some initial studies suggested airway epithelial cells play a crucial role in the defense against pathogens and allergens (Xiao et al., 2011). In a recent study, the authors demonstrated significantly reduced mitochondrial glucocorticoid and estrogen receptors in lung tissue, particularly in human bronchial epithelial cells of fatal asthma patients. These data indicate that excessive ROS released by mitochondria in inflammatory cells might be involved in epithelial cell apoptosis in asthma (Simoes et al., 2012). In line with this, other studies improved our understanding of the role of airway epithelial cells influencing innate immune cells in asthma (Hammad et al., 2009; Rate et al., 2009). Inflammatory cell infiltration plays an integral role in the progression of asthma. Inflammatory cell influx includes the presence of CD4^+^ T helper 2 (Th2) and the CD8^+^ (T cytotoxic) cells along with Th2-associated cytokines. Other cells include eosinophils, macrophages and basophils (Hamid et al., 2003; Locksley, 2010). These studies suggest that finding therapeutic targets that reduces excessive ROS in the immune cells activated by bronchial epithelial cells may prove beneficial. In relevance to mtDNA in asthma, there is no strong evidence to demonstrate the presence of mtDNA mutations in asthma (Raby et al., 2007). However, there are few studies suggesting a connecting link between asthma and mitochondrial abnormalities (Heinzmann et al., 2003; Jones et al., 2004). Taken together, more detailed studies are required to understand the role of mitochondrial dysfunction in the pathogenesis of asthma.

## Mitochondrial dysfunction in cystic fibrosis

Cystic Fibrosis (CF) is a lethal autosomal recessive disease where mutation ΔF508 (loss of phenylalanine residue at position 508) in the gene encoding Cystic Fibrosis Transmembrane Conductance Regulator (CFTR) is responsible for the abnormal mucus secretions (Rowe et al., 2005). There are some studies describing the link between mitochondrial defects, calcium uptake and cystic fibrosis (Feigal and Shapiro, 1979; Shapiro, 1988). In relevance to this, alterations in the pH of mitochondrial complex I were observed in CF patients (Shapiro et al., 1979). On the other hand, downregulation of MT-ND4 expression was observed in CF indicating decreased mitochondrial complex I activity (Chomyn, 2001). Furthermore, air borne particulate matters exposure showed decreased Δψm, elevated ROS generation and increased epithelial cell apoptosis in CF bronchial epithelium (Kamdar et al., 2008). These studies suggest that mitochondrial dysfunction is a direct consequence of CFTR failure.

## Mitochondrial dysfunction in pulmonary arterial hypertension

Pulmonary arterial hypertension (PAH) is a vascular disease caused by hyperproliferation of vascular cells that eventually eliminate the pulmonary arterial lumen and leads to right ventricular failure and premature death. It is interesting to note that mitochondria are hyperpolarized in pulmonary artery smooth muscle cells (PASMCs). So, PASMC mitochondria may serve as possible therapeutic targets for PAH. In one study, silencing MFN2 and a mitochondrial biogenesis marker peroxisome proliferator-activated receptor gamma coactivator 1-alpha (PGC1α) contributed to mitochondrial fragmentation, increased proliferation and impaired apoptosis. Furthermore, overexpression of MFN2 using an adenoviral vector showed increased fusion, reduced proliferation, and increased apoptosis in human PAH (Ryan et al., 2013). In a couple of elegant studies, dichloroacetic acid and malonyl-CoA decarboxylase null mutant mice demonstrated reversal of pulmonary artery modeling and showed complete resistance to the development of PAH induced by hypoxia or monocrotaline in rats and mice models respectively. (McMurtry et al., 2004; Sutendra et al., 2010). These observations indicate that mitochondrial dysfunction plays a key role in the diathesis of PAH.

## Conclusions

The literature discussed in this review article reemphasizes the complexity of mitochondrial biology especially in relation to lung diseases. Undoubtedly, healthy maintenance of mitochondrial structure and function is central to normal human physiology. In the past few decades, mitochondrial structure and morphology in normal physiology have been extensively studied. However, mitochondrial biology in relevance to lung diseases is poorly understood. Furthermore, mitochondrial dysfunction caused by aberrant mitochondrial dynamics together with excessive mitochondrial ROS is observed to be an early-onset marker in many lung diseases. Thus, mitochondria play a critical role in coordination between life and death signaling pathways of lung diseases.

It is believed that aberrant mitochondrial dynamics linked with abnormal or compromised mitophagy results in excessive production of mitochondrial ROS inside the cell. This accumulation of dysfunctional mitochondria may lead to apoptosis or necrosis and therefore, contribute to the pathogenesis of lung diseases. In hindsight, lowering mitochondrial ROS to physiological concentrations both in aging and non-aging related lung diseases could result in the cessation of unwarranted downstream signaling pathways. Future studies utilizing humanized mice models that mimic mitochondrial dysfunction are necessary to accelerate the basic biology seeking to understand the pathophysiological mechanisms of lung diseases. Investigating approaches that reverse oxidative modification and inactivation of mitochondrial proteins and maintains mitochondria quality control mechanisms may have significant therapeutic potential for lung diseases.

## Funding

Funded by NIH grant HL 085103 (Vineet Bhandari).

### Conflict of interest statement

The authors declare that the research was conducted in the absence of any commercial or financial relationships that could be construed as a potential conflict of interest.
